# Differentiation of grapevine (*Vitis vinifera* L.) *conculta* members based on molecular tools

**DOI:** 10.1080/13102818.2014.901666

**Published:** 2014-05-06

**Authors:** Peter Bodor, Antal Szoke, Kitti Toth-Lencses, Aniko Veres, Tamas Deak, Pal Kozma, Gyorgy Denes Bisztray, Erzsebet Kiss

**Affiliations:** ^a^ Institute of Viticulture and Enology, Corvinus University of Budapest, Budapest, Hungary; ^b^ Institute of Genetics and Biotechnology, Szent Istvan University, Gödöllő, Hungary; ^c^ Institute of Viticulture and Enology, University of Pecs, Pecs, Hungary

**Keywords:** *conculta*, SSR, *VvMybA1*, *Gret1* retroelement

## Abstract

Twenty-seven grapevine (*Vitis vinifera L.*) varieties within 12 putative berry colour variation groups (*conculta*) were genotyped with 14 highly polymorphic microsatellite (simple sequence repeats (SSR)) markers. Three additional oligonucleotide primers were applied for the detection of the Gret1 retroelement insertion in the promoter region of *VvMybA1* transcription factor gene regulating the UFGT (UDP-glucose: flavonoid 3-O-glucosyltransferase) activity. UFGT is the key enzyme of the anthocyanin biosynthetic pathway. SSR results proved that the analysed cultivars can be grouped only into nine *concultas*, the other three putative berry colour variant groups consist of homonyms as a consequence of misnaming. In the case of Sárfehér-Sárpiros, Delaware red-Delaware white and Járdovány fekete-Járdovány fehér, it was attested that they are not bud sports, but homonyms. Some *conculta* members could be differentiated according to the presence or the absence of the *Gret1* retroelement (Chasselas, Furmint and Lisztes), while others, Bajor, Bakator, Gohér and Traminer *conculta* members, remained indistinguishable either by the microsatellites or the *Gret1*-based method.

## Introduction

Mutations affected important traits of horticultural plants to a great extent during domestication of these species including fruit colour variations.[1] Zhukovsky [2] introduced the term of *conculta* for the colour mutant varieties. The Hungarian ampelographer Márton Németh [3] also adopted and extended this over-cultivar taxonomic category for grapevine. According to his theory, the grapevine *conculta* members originate from blue-berried ancestors as a consequence of bud mutation. The difference between the members can be recognized only by the colour of the berry, the autumn leaf colouration and the prostrate hairs of the shoot tips. Based on morphological traits Németh [4] differentiated 26 *concultas*, grown in Hungary.

The identification of the cultivars is very important at all phenological stages.[5] Besides ampelographic descriptors DNA fingerprints are significant tools in varietal characterization in grapevine. Among these molecular methods microsatellite (SSR = simple sequence repeats) analyses became widely used due to their reliability and high reproducibility.[6–8] Although the SSR markers made it possible to detect even clonal variations,[9–11] they are not applicable in all cases of somaclonal variations like bud mutations.[12] Bowers et al. [6] and Regner et al. [12] concluded that the SSR markers are not suitable for the discrimination of the members of Pinot group (Pinot blanc, Pinot gris, Pinot noir). Other molecular methods like Restriction Fragment Length Polymorphism (RFLP) and Random Amlified Polymorphic DNA (RAPD) also proved to be ineffective for differentiating berry colour variants.[13–16] Halász et al. [14] analysing autochthonous varieties in the Carpathian Basin with six polymorphic SSR markers could not discriminate the members of the Bajor, Bakator, Gohér and Lisztes *concultas.* Additionally they concluded that because of the high allelic differences the cultivar Bakator kék cannot be the member of the Bakator group. The name Bakator is a homonym confirming that the similarity in names does not certainly mean a bud mutation event. Existence of homonyms and synonyms is very frequent in the nomenclature.[8,17]

Slinkard and Singleton [18] suggested that the white berried cultivars originate from the coloured ones by loss-of-function mutations. The berry colour is determined by anthocyanin accumulation in the skin, which varies greatly in concentration and composition depending on the grape cultivar.[19] The key enzyme of anthocyanin biosynthesis is the UDP-glucose-flavonoid 3-O-glucosyl-transferase (UFGT). This enzyme does not express in the white berried cultivars, in spite of the fact that there are no differences in both *VvUFGT* promoter and coding region between white and coloured cultivars.[19,20] The anthocyanin biosynthesis is controlled by a transcription complex including the *Myb* genes, which activates the *UFGT* gene.[21]

The ancient wild grape had coloured berries and the nowadays-cultivated varieties derived from the ancient form. The white cultivars arose mostly from red-berried parents by different mutations in two adjacent *Myb* genes, *VvMybA1* and *VvMybA2*.[22,23] Among these mutations, insertion of a retroelement, the *Gret1* retrotransposon into the promoter region of the *VvMybA1* gene leading to transcriptional inactivation of *VvMybA1* was first identified in cultivars Italia and Muscat of Alexandria.[22] This mutant allele was named *VvMybA1a*, while the functional allele of the coloured cultivars is *VvMybA1c.* White berried cultivars are homozygous for *Gret1* insertion, whereas the colour-skinned varieties contain at least one functional allele. In several white cultivars deletion of *Gret1* from promoter region was observed resulting in a functional allele, *VvMybA1b*, containing only a short part, the 3′-LTR region of the retrotransposon, thus these types of red cultivars derived from their white-skinned progenitor.[22,23] Single nucleotide polymorphism in *VvMybA2* coding region also could result in white berries.[11] Based on the results of Mitani et al. [24] wild *Vitis* species do not carry the *VvMybA1* locus even if they are white berried.

Yakushiji et al. [25] showed that the deletional mutation of functional *VvMybA1c* from Pinot noir resulted in Pinot blanc. At the same time the other members of the Pinot *conculta*, Pinot gris and Pinot noir are undistinguishable with the retroelement based method.[26]

Kobayashi et al. [20] and Giannetto et al. [26] concluded that the colour mutations can be bidirectional: black-to-white and white-to-red/pink between the *conculta* members. These facts confirmed the result of Walker et al. [23] identifying two pale coloured mutations of the Cabernet sauvignon (Malian and Shalistin) as a deletion consequence in two regulatory genes of the berry colour locus.

In this paper we combine the knowledge of the cultivar characterization with SSR markers and the application of the *Gret1* retroelement for discriminating 16 local Hungarian and 11 international putative or already proven bud sports.

## Materials and methods

### Plant material

The cultivars investigated in this study are listed in Table 2. Young leaves of the grapevine varieties were collected from grapevine collections maintained at Károly Róbert College in Eger, Szőlőskert Ltd. in Nagyréde, Helvécia and University of Pécs, Institute of Viticulture and Enology.

**Table 1.** SSR allele sizes of the 27 cultivars at 14 loci.

Accession name	Berry colour	Scu10	VVS2	VVMD5	VVMD7	VVMD21	VVMD25	VVMD27	VVMD28	VVMD31	VVMD36	VrZAG62	VrZAG79	VrZAG83	VrZAG112
Bajor feketefájú	Black	202:208	134:154	228:238	243:243	248:256	242:244	182:196	238:250	209:209	254:254	192:200	252:262	193:193	243:243
Bajor kék	Black	202:208	134:154	228:238	243:243	248:256	242:244	182:196	238:250	209:209	254:254	192:200	252:262	193:193	243:243
Bajor szürke	Grey	202:208	134:154	228:238	243:243	248:256	242:244	182:196	238:250	209:209	254:254	192:200	252:262	193:193	243:243
Bakator piros	Red	202:208	134:134	228:242	243:257	242:256	244:244	180:186	236:250	201:209	266:288	192:198	254:254	191:197	237:241
Bakator tüdőszínű	Pink	202:208	134:134	228:242	243:257	242:256	244:244	180:186	236:250	201:209	266:288	192.198	254:254	191:197	237:241
Chasselas blanc	White	204:214	134:144	228:236	242.250	248:266	244:260	186:190	220:270	209:213	264:264	196:206	252:260	193:203	243:243
Chasselas rouge	Red	204:214	134:144	228:236	242.250	248:266	244:260	186:190	220:270	209:213	264:264	196:206	252:260	193:203	243:243
Delaware fehér	White	202:204	134:134	228:228	239:243	242:266	244:244	186:190	234:248	203:209	258:264	198:208	252:258	191:203	243:243
Delaware piros	Red	204:210	146:150	236:236	231:231	220:220	244:250	204:210	218:254	199:201	240:250	202:212	252:260	159:167	231:243
Furmint fehér	White	202:208	134:154	228:242	243:253	248:258	242:244	180:196	230:250	209:209	254:276	192:208	238:250	191:191	243:243
Furmint piros	Red	202:208	134:154	228:242	243:253	248:258	242:244	180:196	230:250	209:209	254:276	192:208	238:250	191:191	243:243
Gohér fehér	White	202:208	134:154	240:240	243:253	242:256	242:244	182:196	236:250	207:211	254:288	190:208	252:262	193:193	241:241
Gohér piros	Red	202:208	134:154	240:240	243:253	242:256	242:244	182:196	236:250	207:211	254:288	190:208	252.262	193:193	241:241
Gohér változó	White	202:208	134:154	240:240	243:253	242:256	242:244	182:196	236:250	207:211	254:288	190:208	252:262	193:193	241:241
Járdovány fehér	White	208:214	142:144	228:236	243:253	242:248	242:244	180:196	230:262	209:209	266:276	200:208	250:250	197:197	245:245
Járdovány fekete	Black	202:208	134:154	222:232	243:253	248:258	242:244	180:196	230:250	209:209	254:276	200:208	250:250	191:191	237:245
Lisztes fehér	White	208:208	134:144	228:234	251:251	248:256	242:244	182:182	236:250	207:211	276:288	208:208	238:260	193:197	241:241
Lisztes piros	Red	208:208	134:144	228:234	251:251	248:256	242:244	182:182	236:250	207:211	276:288	208:208	238:260	193:197	241:241
Merlot	Black	202:216	140:152	228:238	243:251	242:248	242:254	190:192	230:236	209:213	254:254	198:198	260:260	197:203	231:245
Merlot gris	Grey	202:216	140:152	228:238	243:251	242:248	242:254	190:192	230:236	209:213	254:254	198:198	260:260	197:203	231:245
Pinot blanc	White	204:216	138:152	230:240	243:247	248:248	242:252	186:190	220:236	213:213	254:254	192:198	242:248	191:203	243:243
Pinot gris	Grey	204:216	138:152	230:240	243:247	248:248	242:252	186:190	220:236	213:213	254:254	192:198	242:248	191:203	243:243
Pinot noir	Black	204:216	138:152	230:240	243:247	248:248	242:252	186:190	220:236	213:213	254:254	192:198	242:248	191:203	243:243
Sárfehér	White	202:208	134:152	226:238	251:259	242:248	244:258	182:196	250:280	207:207	264:264	208:208	250:250	191:193	245:245
Sárpiros	Red	202:208	134:134	226:240	251:257	242:242	242:258	180:196	270:280	207:209	264:288	190.208	250:258	193:193	237:245
Traminer	White	204:208	152:152	234:240	247:261	248:248	254:254	190:190	236:236	201:213	254:264	192:198	248:254	191:203	237:243
Traminer red	Red	204:208	152:152	234:240	247:261	248:248	254:254	190:190	236:236	201:213	254:264	192:198	248:254	191:203	237:243

**Table 2.** The analysed cultivars with the site of collection, geographical origin according to Németh,[4] berry colour, the possibility of discrimination by SSR method or by the *Gret1* insertion into the *VvMybA1* locus.

				Possibility of discrimination with	
Accession name	Collection site	Geographical origin	Berry colour	SSR	*VvMybA1*
Bajor feketefájú	Pécs	*convar. pontica*	Black	No	no
Bajor kék	Pécs	*convar. pontica*	Black	No	yes
Bajor szürke	Pécs	*convar. pontica*	Grey	No	no
Bakator piros	Pécs	*convar. pontica*	Red	No	No
Bakator tüdőszínű	Pécs	*convar. pontica*	Pink	No	No
Chasselas blanc	Nagyréde	*convar. orientalis*	White	No	Yes
Chasselas rouge	Nagyréde	*convar. orientalis*	Red	No	Yes
Delaware red	Eger	*hybrid*	Red	Yes	Yes
Delaware white	Eger	*hybrid*	White	Yes	Yes
Furmint fehér	Pécs	*convar. pontica*	White	No	Yes
Furmint piros	Pécs	*convar. pontica*	Red	No	Yes
Gohér fehér	Pécs	*convar. pontica*	White	No	No
Gohér piros	Pécs	*convar. pontica*	Red	No	No
Gohér változó	Pécs	*convar. pontica*	White	No	No
Járdovány fehér	Helvécia	*convar. pontica*	White	Yes	Yes
Járdovány fekete	Helvécia	*convar. pontica*	Black	Yes	Yes
Lisztes fehér	Pécs	*convar. pontica*	White	No	Yes
Lisztes piros	Pécs	*convar. pontica*	Red	No	Yes
Merlot	Pécs	*convar. occidentalis*	Black	No	No
Merlot gris	Pécs	*convar. Occidentalis*	Grey	No	No
Pinot blanc	Nagyréde	*convar. occidentalis*	White	No	Yes
Pinot gris	Nagyréde	*convar. occidentalis*	Grey	No	No
Pinot noir	Nagyréde	*convar. occidentalis*	Black	No	No
Sárfehér	Helvécia	*convar. pontica*	White	Yes	Yes
Sárpiros	Helvécia	*convar. pontica*	Red	Yes	Yes
Traminer	Eger	*convar. occidentalis*	White	No	No
Traminer red	Eger	*convar. occidentalis*	Red	No	No

Note: The Hungarian names indicate the berry colour of the cultivars: fehér = white; piros = red; változó = altering; szürke = grey; fekete = black, tüdőszínű = pink.

### Short characterization of the putative *concultas*

*Bajor* is probably a Hungarian variety. It used to be cultivated in most wine regions of Hungary; presently it has no importance in the Hungarian viticulture – it can be found only in old plantations of quality wine regions (Tokaj Hegyalja, Mecsek).[4,27]

*Bakator* is a Carpathian Basin variety. Kék bakator, Piros, Tüdőszínű and Fehér Bakator carry the Bakator name; however, it was shown by SSR analysis that Kék bakator is a different cultivar and not a berry colour variant of Bakators.[14] Nowadays only Bakator piros is cultivated in Hungary on a few hectares.[27]

*Chasselas*: in spite of its French name it derives from Asia, its way to Europe is unknown. The most widely cultivated table grape in Hungary, Chasselas rouge, rose and b1ancs are the members of the *conculta.*[4,27]

*Delaware red* is of North-American origin, assumably a natural hybrid of *Vitis vinifera*, *Vitis labrusca* and *Vitis aestivalis*. In Hungary it can be found only in old vine yards, however earlier it was a popular cultivar in the Trans-Danubian part of Hungary. Delaware white was supposed to be the progeny of Delaware red as a result of open pollination.[27]

*Furmint*: old Hungarian wine grape with three variants: piros, fehér, változó. Fehér Furmint is the third most widespread cultivar in Hungary, particularly in the Tokaj Hegyalja region, one of the components of the world famous Tokay aszu. Piros and változó Furmints are maintained in gene banks.[27,28]

*Gohér*: old Hungarian variety, used as table and wine grape in the past, it has three variants (fehér, piros, változó). Recently Gohér fehér has been planted at the famous Tokaj wine region. Gohér piros and változó are conserved in gene banks.[27,28]

*Járdovány*: its origin is unknown. It was widespread in the past, but nowadays it can be found mainly in old plantations. Járdovány fehér wine is not a characteristic one, but Járdovány fekete has better quality, therefore it has got permission to be planted as wine grape in some regions of Hungary.[27]

*Lisztes*: old autochthonous variety of the Carpathian Basin, high yield and low wine quality are characteristic of Lisztes; it has no role in the present Hungarian viticulture.[4]

*Merlot*: known since the eighteenth century, derives from France (Bordeaux). It is cultivated all over the world; it was registered in Hungary in 1973.[27,28]

*Pinot* derives from France, where it has been growing for centuries. It is a worldwide cultivated variety with the following berry colour variants: Pinot gris, noir, blanc, rose and violet.[27]

*Sárfehér* is an old white berried Hungarian cultivar. Because of the first syllable of its name (Sár = mud) it can be assumed that the red berried Sárpiros is also a berry colour variant. Before the *Phylloxera* epidemic it was the characteristic variety of Somló and Neszmély in Hungary.[27,28]

*Traminer*: its origin is uncertain. Generally it was thought to derive from Tramin (village in South Tirol). Red, blue and white berry colour variants of Traminer are known, but only the red grape is cultivated in Hungary, the other two exist only in collections.[27]

### DNA extraction and Polymerase Chain Reaction (PCR) analysis

DNA was extracted with Qiagen DNeasy Plant Mini Kit according to the manufacturer's protocol (Qiagen-Biomarker Ltd., Gödöllő, Hungary). The quality and quantity of the isolated DNA was checked on 1.5% agarose gel with electrophoresis and by NanoDrop spectrophotometer (BioScience Ltd. Budapest).

Fourteen fluorescent-labelled (FAM-6 and Cy5) microsatellite primer pairs: Scu10, VVS2,[29] VVMD5, VVMD7,[30] VVMD21, VVMD25, VVMD27, VVMD28, VVMD31, VVMD36,[31] VrZag62, VrZag79, VrZag83 and VrZag112 [32] were used in the analyses. The PCR conditions are described by Regner et al. [12] and Halász et al.[14]

For the detection of the *Gret1* retroelement in the promoter region of the *VvmybA1* three oligonucleotide primers were used as reported by Kobayashi et al.,[33] with the modification of This et al.[34] The PCRs were carried out in an iCycler (BioRad) equipment. The PCR program for the amplification of *Gret1* was as follows: initial denaturation at 94 °C for 3 min, 35 cycles of 94 °C for 30 sec/55 °C for 30 sec/72 °C for 90 sec, with a final step at 72 °C for 10 min. PCR products were checked after 1.2% agarose gel electrophoresis. SSR loci were analysed on ABI 310 genetic analyzer (Biomi Ltd., Gödöllő, Hungary) and ALF-Express DNA Fragment Analyzer (Amersham Biosciences, AP Hungary Ltd., Budapest, Hungary).

The standardization of the allele sizes was made by using French and Hungarian reference varieties such as Pinot noir, Chardonnay and Irsai Olivér, Csaba gyöngye, and Pozsonyi, respectively. Among these cultivars some parent-progeny relationships exist confirmed by SSR results.[31,35]

## Results and discussion

### SSR analysis

The SSR profiles of the characterized 27 samples are presented in Table 1. The SSR analysis resulted in 15 different allele profiles including nine *concultas*. Individual varietal differences could be observed in the case of the following cultivars: Delaware white-Delaware red, Sárfehér-Sárpiros and Járdovány fehér-Járdovány fekete meaning that they do not constitute *concultas*, it can be supposed that these cultivars have only homonym names and they are not the results of bud mutations. Homonymy and synonymy is a common phenomenon in grapevine nomenclature, which can be clarified by microsatellite markers.[36–38]

At the same time based on 14 microsatellite loci there were no differences in allele sizes within the following *concultas*: Bajor, Bakator, Chasselas, Furmint, Gohér, Lisztes, Merlot, Pinot and Traminer (Table 1). Our results supported the earlier conclusions of Regner et al. [12] that there are no SSR allele differences between Pinot samples.

Two members of the Chasselas, Merlot and Traminer group were analysed in this work. Based on the SSR profiles, none of the putative *conculta* members differed from each other at the investigated loci.

According to Galet [39] and Csepregi and Zilai [28] Delaware white is the seedling of Delaware red (*V. labrusca* L. × *V. aestivalis* Michx.) but their SSR profile excludes the possibility of parent–progeny relationship. These cultivars are homonyms and not the results of either bud mutation or paternity.

In the case of the cultivars Sárfehér-Sárpiros and Járdovány fekete-Járdovány fehér the name identity can be explained also with homonymy since the genetic profile disclaims the possibility of colour mutation.

These results suggest the following conclusion: similar or identical names can originate from two main sources: (1) emergence of *concultas* as a consequence of bud mutation (Bajor, Bakator, Chasselas, Furmint, Gohér, Lisztes, Merlot, Pinot and Traminer) and (2) homonymy (Delaware red-Delaware white, Járdovány fekete-Járdovány fehér, Sárfehér-Sárpiros).

### Molecular analyses of *VvMybA1* locus

Since *conculta* members were indistinguishable by SSR markers our second aim was not only to differentiate them but also find the reason for the berry colour variation. The method that we applied is based on detecting the presence or absence of the *Gret1* retrotransposon in the promoter region of the *VvMybA1* transcription factor gene. In majority of the white berried cultivars transcription of the *UFGT* gene (coding the key enzyme in the anthocyanin biosynthetic pathway) is blocked, because of the insertion of *Gret1* retrotransposon into *VvMybA1* promoter.[25,34] This et al. [34] reported three white berried cultivars (Avgoulato, Gamay Castille mutation blanche and Sultanina-Gora Chirine) which did not contain the *Gret1* in the *VvMybA1* promoter.

Kobayashi et al. [22] described three alleles at *VvMybA1* locus based on the presence (*VvMybA1a*) or absence of *Gret1* (*VvMybA1c*) element in the promoter of the gene. The third one *VvMybA1b* contains a 3′-LTR sequence remaining after *Gret1* deletion.

**Figure 1.**
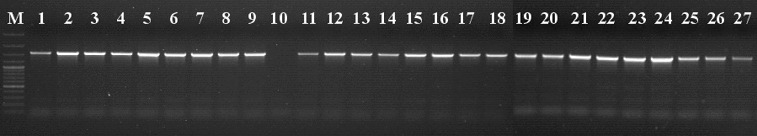
Detection of *VvMybA1a* allele (containing *Gret1* retrotransposon). M: DNA molecular weight marker (Fermentas GeneRuler 100 bp Ladder Plus / 3000 bp, 2000 bp, 1500 bp, 1200 bp, 1031 bp, 900 bp, 800 bp, 700 bp, 600 bp, 500 bp, 400 bp, 300 bp, 200 bp, 100 bp); Lane 1: Lisztes piros; Lane 2: Lisztes fehér; Lane 3: Bakator piros; Lane 4: Bakator tüdőszínű; Lane 5: Gohér piros; Lane 6: Gohér fehér; Lane 7: Gohér változó; Lane 8: Furmint piros; Lane 9: Furmint fehér; Lane 10: Bajor kék; Lane 11: Bajor feketefájú; Lane 12: Bajor szürke; Lane 13: Sárpiros; Lane 14: Sárfehér; Lane 15: Járdovány fekete; Lane 16: Járdovány fehér; Lane 17: Chasselas rouge; Lane 18: Chasselas blanc; Lane 19: Pinot noir; Lane 20: Pinot gris; Lane 21: Pinot blanc; Lane 22: Traminer red; Lane 23: Traminer; Lane 24: Merlot; Lane 25: Merlot gris; Lane 26: Delaware red; Lane 27: Delaware white.

**Figure 2.**
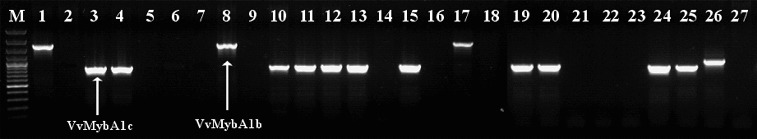
Detection of *VvMybA1b* and *VvMybA1c* alleles. M: DNA molecular weight marker (Fermentas GeneRuler 100 bp Ladder Plus / 3000 bp, 2000 bp, 1500 bp, 1200 bp, 1031 bp, 900 bp, 800 bp, 700 bp, 600 bp, 500 bp, 400 bp, 300 bp, 200 bp, 100 bp); Lane 1: Lisztes piros; Lane 2: Lisztes fehér; Lane 3: Bakator piros; Lane 4: Bakator tüdőszínű; Lane 5: Gohér piros; Lane 6: Gohér fehér; Lane 7: Gohér változó; Lane 8: Furmint piros; Lane 9: Furmint fehér; Lane 10: Bajor kék; Lane 11: Bajor feketefájú; Lane 12: Bajor szürke; Lane 13: Sárpiros; Lane 14: Sárfehér; Lane 15: Járdovány fekete; Lane 16: Járdovány fehér; Lane 17: Chasselas rouge; Lane 18: Chasselas blanc; Lane 19: Pinot noir; Lane 20: Pinot gris; Lane 21: Pinot blanc; Lane 22: Traminer red; Lane 23: Traminer; Lane 24: Merlot; Lane 25: Merlot gris; Lane 26: Delaware red; Lane 27: Delaware white.

The results of PCR analysis of the promoter region of the *VvMybA1* transcription factor gene in five proven Hungarian *concultas* (Bajor, Bakator, Furmint, Gohér and Lisztes) are shown in Figure 1. Amplification of an ∼1500 bp DNA fragment indicates the presence of *VvMybA1a* allele in each variety (Figure 1), independently of the actual berry colour except Bajor kék which does not contain the *Gret1* insertion at all (it is homozygous for the *VvMybA1c* allele). Analysis of the functional *VvMybA1* allele resulted in PCR products only in the coloured cultivars due to *VvMybA1b* and *VvMybA1c* alleles (Figure 2). The coloured members of these five Hungarian *concultas*, Bajor szürke, Bajor feketefájú, Bakator piros Bakator tüdőszínű, Furmint piros and Lisztes piros are heterozygous for the *Gret1* retroelement, therefore the anthocyanin biosynthesis is undisturbed (Figure 2). Interestingly the red berried Gohér piros does not contain any functional *VvMybA1* (*b*, *c*) alleles. Two members of Bajor *conculta* (Bajor szürke and feketefájú) gave identical DNA pattern at the *VvMybA1* locus, showing that they contain both the functional *VvMybA1c* and the non-functional *VvMybA1a* alleles.

Our results are in accordance with the earlier published works on the correlation between the presence of *Gret1* and the loss of the berry colour.[26,34]

Although Sárfehér, Járdovány fekete and Delaware red have not proven to be *concultas*, we analysed their *VvMybA1* locus. Red berried Sárpiros and Járdovány fekete are heterozygous for the *Gret1* insertion.

Surprisingly, in the case of the characterization of the promoter region of the *VvMybA1* transcription factor gene, the primers amplified a larger fragment in the same region in Delaware red (Figure 2).

In Traminer red – similar to Gohér piros – no functional *VvMybA1b* or *c* alleles could be detected. The members of the Bajor, Bakator and Gohér *concultas* were indistinguishable based on the *Gret1* retroelement. Thus these data call for a further study in order to find an unambiguous method for distinguishing varieties in the *concultas*. Our results also revealed that Chasselas rouge – likewise Lisztes piros and Furmint piros – contains a *VvMybA1b* allele. The possibility of discrimination and identification of the investigated cultivars are listed in Table 2.

## Conclusions

The results of the present study demonstrate that the applied 14 SSR markers were appropriate to prove which cultivars constitute *concultas*. Nine of the 12 putative cultivar groups can be considered as real *concultas*. Members of these *concultas* were indistinguishable with the SSR primer set used in this study. Earlier results proved the loss of berry colouration to be the consequence of *Gret1* insertion in the *VvMybA1* promoter region. Therefore we analysed the *VvMybA1* locus coding a key transcriptional factor of berry anthocyanin biosynthesis. Testing three alleles of *VvMybA1* (*a*, *b*, *c*) among the five Hungarian *concultas* only Furmint piros and Lisztes piros could be differentiated from the white berried variants. Bajor (except Bajor kék), Bakator and Gohér *concultas* require further investigations to clarify the genetic background of their berry colour.
